# Preliminary Reference Values for Plantar Fat Pad Thickness Beneath the Metatarsal Heads and Its Relationship with Body Mass Index

**DOI:** 10.3390/healthcare13243219

**Published:** 2025-12-09

**Authors:** Raquel Sánchez-Rodríguez, Andrés Ponce-Barrero, Marina Fontán-Jiménez, María Victoria Cáceres-Madrid, Raquel Fragua-Blanca, Víctor García-Maqueda

**Affiliations:** 1 Department of Nursing, University of Extremadura, 10600 Plasencia, Spain; marinaf@unex.es (M.F.-J.); pgvicky@unex.es (M.V.C.-M.); victorgm@unex.es (V.G.-M.); 2 Clínica del pie Mónica Sarasa, Av. Pamplona, 10, Bajo, 31010 Barañain, Spain; poncebarreoa@outlook.com; 3 Department of Nursing, Physioterapy and Occupational Therapy, Faculty of Health Sciences, University of Castilla-La Mancha, 45600 Talavera de la Reina, Spain; raquel.fragua@uclm.es; 4 Clínica Podovite, C/Jesús Rasero 17, 06300 Zafra, Spain

**Keywords:** ultrasonography, plantar fat pad, forefoot, metatarsal heads, body mass index

## Abstract

**Introduction and Objectives:** The thickness of the plantar fat pad (PFP) beneath the metatarsal heads may play a protective role in preventing forefoot disorders such as metatarsalgia. However, reference values for plantar adipose tissue thickness in this region among healthy individuals are currently unavailable. Therefore, the aim of this study was to determine, by means of ultrasound imaging, the thickness of the PFP beneath the five metatarsal heads and to analyze its possible relationship with body mass index (BMI). **Materials and Methods**: Thirty-five young adults (17 males and 18 females) with neutral feet, free from deformities or pain, participated in the study. Using a VINNO E35 ultrasound device, the thickness of the PFP beneath each of the five metatarsal heads was quantified. A linear transducer was positioned longitudinally along the axis of each metatarsal. The distance between the dermis and the flexor tendon was measured from the second to the fifth metatarsal heads, and from the fibular sesamoid for the first metatarsal head. **Results:** The central forefoot showed the greatest PFP thickness (2nd metatarsal head, 7.1 ± 0.9 mm; 3rd metatarsal head, 6.9 ± 0.9 mm). No significant differences in PFP thickness were found between sexes. However, a positive correlation was observed between BMI and PFP thickness at the fourth metatarsal head (r = 0.358, *p* = 0.035). **Conclusions:** The study demonstrated greater PFP thickness beneath the second and third metatarsal heads, with no significant sex-related differences. These findings indicate a consistent anatomical pattern independent of sex in young, healthy individuals. Moreover, a moderate influence of BMI was identified at the fourth metatarsal head, which could represent a potential protective mechanism against forefoot overload.

## 1. Introduction

The fat pad is a connective and adipose layer located between the reticular dermis and deeper structures such as the plantar fascia, muscles, and bones [1]. In the forefoot, beneath the metatarsal heads, the plantar fat pad is organized into closed compartments with a honeycomb-like structure, where adipocytes are surrounded by fibrous septa rich in elastin. These septa, composed of parallel collagen fibrils, are firmly anchored to the skin and adjacent bones, providing stability and resistance to compression [2,3]. In addition, this structure contains blood vessels and nerve endings, including Pacinian corpuscles, which contribute to plantar sensory function [3,4]. Owing to this structural arrangement, the PFP performs essential physiological and biomechanical roles, including thermal regulation, protection against impact, and acting as a shock-absorbing system that facilitates the absorption and distribution of plantar loads during gait and other weight-bearing activities [5]. A reduction in the thickness of this tissue has been strongly associated with the development of plantar ulcers, as the capacity to absorb ground reaction forces becomes compromised [4,6].

Several factors may influence the thickness and distribution of the PFP. In the heel, men have been shown to exhibit greater PFP compared with women [7,8], and individuals with higher BMI values tend to have a thicker plantar adipose layer [9,10]. Age also plays a relevant role, with maximal thickness reported between 30 and 44 years, followed by a progressive decline [8]. The reduction in this tissue in the heel has been associated with painful conditions such as plantar fasciitis [11] and heel fat pad syndrome, characterized by fat pad atrophy and chronic heel pain [12,13].

Regarding the forefoot, the relationship between PFP thickness and metatarsal pathology remains controversial. Different studies have reported variability in measurements, as some assess the total soft-tissue thickness (from the skin to the metatarsal head), whereas others focus specifically on the adipose layer. Waldecker [14] concluded that alterations in PFP thickness were not directly related to the frequency or intensity of metatarsal pain. Similarly, digital deformities such as claw toes or hallux valgus have not shown a clear correlation with the reduction in PFP beneath the metatarsal heads [15].

It is worth noting that previous research has employed heterogeneous methodologies, measuring PFP at different locations (e.g., under the second and third metatarsal heads or under the first and fifth), without consistently evaluating all metatarsal heads. Moreover, studies assessing plantar fat thickness beneath all five metatarsal heads have primarily focused on diabetic populations, limiting the generalization of findings to healthy individuals.

Currently, there are no reference values available for PFP thickness in the forefoot area measured under non-weight-bearing conditions in young, healthy individuals, nor is there any information on its possible relationship with sex or BMI. This information is essential, as this tissue is the main structure responsible for cushioning plantar compressive forces and may play an important role in preventing foot pathologies when its thickness is reduced below normal values. In this context, analyzing the thickness of the plantar adipose pad in young, healthy subjects provides key data for establishing reference values that can serve as a basis for clinical evaluation and early detection of functional alterations. Furthermore, characterizing the thickness of this tissue in young adults is fully in line with the objectives of contemporary healthcare, as it provides normative data that can contribute to the development of preventive care strategies, the optimization of physical performance, and the promotion of musculoskeletal health from the earliest stages of life.

Therefore, the aim of the present study is to determine the thickness of the PFP beneath the five metatarsal heads in a sample of young, healthy subjects, and to analyze potential differences according to sex and BMI.

## 2. Materials and Methods

### 2.1. Participants

The study was conducted at the Podiatric Clinic of the University of Extremadura in Plasencia (Spain) and was approved by the University’s Bioethics and Biosafety Committee (ID: 56/2024). Written informed consent was obtained from all participants prior to inclusion in the study. The sample consisted of 35 subjects (17 males and 18 females). The mean age was 22.71 ± 2.27 years (range: 18–27), and the mean BMI was 24.19 ± 2.89 kg/m^2^ (range: 19.8–32). The remaining anthropometric characteristics by sex are presented in Table 1.

Inclusion criteria for participation in the study were: (a) age between 18 and 30 years, and (b) absence of metatarsalgia or hyperkeratosis. Exclusion criteria included: (a) a history of previous lower-limb surgery; (b) presence of pain in the lower limbs; (c) presence of foot deformities (hallux valgus, hallux rigidus, claw toes, etc.) and (d) any other forefoot or digital dealignments (i.e., clinodactylies)

### 2.2. Procedure

A podiatrist with more than 20 years of clinical experience in the management of foot disorders collected the anthropometric variables (age, sex, and BMI), screened participants for inclusion and exclusion criteria, and performed the measurements of PFP thickness at the level of the five metatarsal heads.

**Figure 1:**
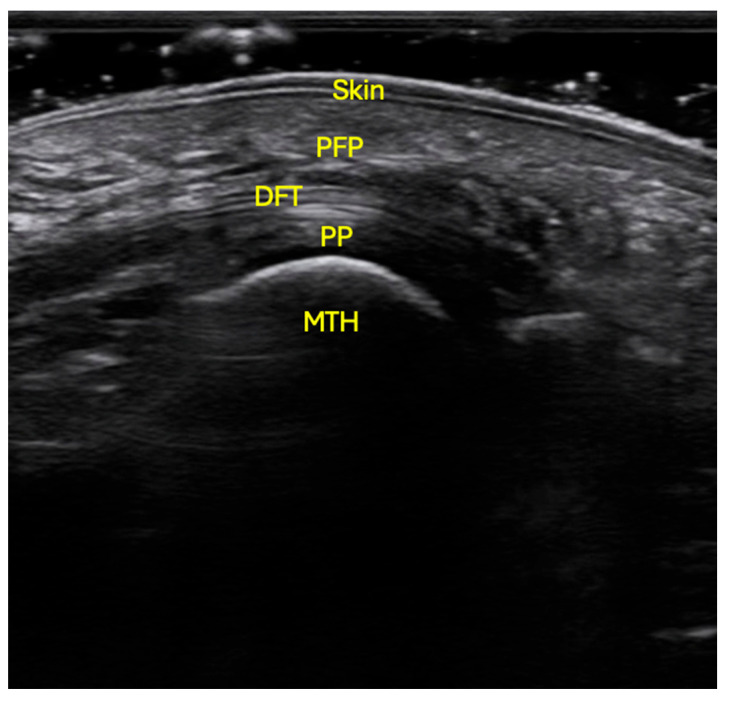
Ultrasonographic localization of the PFP at the level of the 2nd to 5th metatarsal heads. The probe was situated in longitudinal orientation, with no pressure, following the axis of the metatarsal, with the patient in prone position, flexed knee and neutral position of the ankle. Skin; PFP: plantar fat pad; DFT: digital flexor tendons; PP: plantar plate; MTH: metatarsal head.

Ultrasound examinations were conducted using a VINNO E35 system (Vinno Technology, Suzhou, China) equipped with a linear transducer model X6-16L, operating at a sampling frequency of 7.3–18 MHz. Participants were positioned prone on the examination table, with the ankle joint in a neutral position and the knee flexed at 90° [16,17]. The skin was cleansed with alcohol to optimize ultrasound penetration. Conductive gel was applied to both the transducer and the plantar surface of the forefoot. The probe was placed longitudinally, aligned with the axis of each metatarsal, without exerting pressure, to obtain a sagittal image free of soft-tissue distortion (Figure 1).

Proper alignment of the transducer was confirmed by visualizing a clear image of the metatarsal cortex and by dynamically moving the corresponding toe with the examiner’s free hand. Once the structures were accurately identified, images were captured from the second to the fifth metatarsal heads. For measurement of PFP thickness, the point of maximum cortical convexity of each metatarsal head was used as a reference, and the distance between the dermis and the flexor tendon was measured. Following the measuring protocol of Morrison et al. for the first metatarsal, the fibular sesamoid bone was used as the anatomical landmark [18]. The probe was initially positioned transversely to locate both sesamoid bones; once the fibular sesamoid was identified, the transducer was rotated 90° to align it longitudinally with the metatarsal axis [18], and the image was captured to measure the PFP thickness from the sesamoid to the dermis. A total of four measurements were obtained for each region of interest.

### 2.3. Statistical Analysis

To ensure data independence, only variables from the participants’ right feet were analyzed, which were randomly selected [19]. The value of PFP thickness for each metatarsal head was calculated as the mean of the four measurements obtained for each region of interest. Statistical analyses were performed using SPSS software version 29.0 for Windows (University of Extremadura, campus license). Descriptive statistics (mean ± SD) were used to determine PFP thickness values. The relationship between sex and tissue thickness was analyzed using the independent samples *t*-test. Pearson’s correlation coefficients were calculated to determine the relationship between BMI and PFP thickness. A *p*-value less than 0.05 was considered statistically significant.

## 3. Results

The greatest PFP thickness was observed beneath the second metatarsal head, with a mean value of 7.1 ± 0.9 mm (Table 2), followed by the third (6.9 ± 0.9 mm) and fourth (6.8 ± 0.9 mm) metatarsal heads. Conversely, the fifth metatarsal head exhibited the lowest thickness, with a mean of 5.8 ± 0.9 mm.

When analyzing differences in PFP thickness between men and women, no significant differences were found at any of the metatarsal heads (Table 3, *p* > 0.05 in all cases).

A moderate positive correlation was found between BMI and the thickness of the PFP beneath the fourth metatarsal head (r = 0.358, *p* = 0.035). No significant correlations were observed for the remaining metatarsal heads, although r values close to 0.315 were noted for the second and third metatarsal heads, with *p*-values of 0.067 and 0.066, respectively, which did not reach statistical significance (Table 4).

## 4. Discussion

The results of the present study indicate that the greatest thickness of the PFP was located beneath the second metatarsal head, followed in decreasing order by the third, fourth, first, and fifth metatarsal heads. This finding can be explained by considering two main aspects: on the one hand, the functional anatomy of the plantar adipose tissue, and on the other, the distribution of forefoot loads during gait.

Plantar adipose tissue is not homogeneously distributed; instead, it is organized into specialized compartments delineated by fibrous septa, whose main function is to cushion and redistribute the loads transmitted to the foot during walking [5]. In this regard, the higher concentration of tissue beneath the second and third metatarsal heads may be interpreted as a protective adaptation to the mechanical demands experienced in this region.

Several plantar pressure analyses have demonstrated that the second and third metatarsal heads represent the key load-bearing areas of the forefoot during the terminal stance and propulsion phases of gait [20,21,22]. This distribution pattern would explain the greater thickness found under these metatarsal heads, in contrast to the thinner tissue observed under the first and fifth heads. Although the first metatarsal head plays a relevant role in final propulsion, the support provided by the sesamoid bones and the plantar capsuloligamentous complex [23] may partially compensate for the need for a thicker adipose layer.

From a clinical perspective, the observed pattern of tissue thickness aligns with the higher prevalence of metatarsalgia and overload injuries reported at the central forefoot. Progressive reduction or atrophy of this PFP—associated with aging, trauma, or metabolic disorders—can compromise its cushioning capacity and increase the susceptibility of this region to injury. Nevertheless, it is noteworthy that the greater PFP thickness identified beneath the second and third metatarsal heads does not correspond to lower plantar pressures, as these areas typically register the highest-pressure values. This finding suggests that forefoot biomechanics in patients with mechanical metatarsalgia may be more strongly influenced by load distribution and central forefoot dynamics than by the absolute thickness of the PFP itself.

When analyzing the distribution of PFP in the forefoot, Kumar et al. [24] described a pattern like that observed in the present study, identifying the second and third metatarsal heads as the thickest regions. In their research, Kumar reported mean thickness values of 6.5 mm for the second and 6.3 mm for the third metatarsal head, while our study obtained slightly higher mean values of 7.1 mm and 6.9 mm, respectively (Table 2), representing a difference of 0.6 mm. This minimal variation could be attributed to differences in participant age, since the mean age in Kumar’s study was 54.47 ± 9.86 years compared to 22.71 ± 2.27 years in our sample. It is important to note that age may also be associated with qualitative degeneration of the fat pad, due to changes in its macro-structural properties (e.g., increased stiffness, loss of elastic septa).

One of the main challenges encountered during this study was the limited availability of prior research analyzing the thickness of the PFP in isolation and under non-weight-bearing conditions, specifically beneath the five metatarsal heads in healthy subjects. Most existing studies have focused on pathological populations, such as those with diabetes mellitus [25,26,27], have assessed plantar fat pad under weight-bearing conditions [16,17,25,26,28], or have evaluated the total plantar soft tissue complex [16,25,26,27,29], making direct comparisons difficult. Accordingly, the present study aims to establish non-weight-bearing preliminary reference values to aid in the clinical interpretation of PFP thickness in preventive and biomechanical assessment contexts for healthy individuals. Although these measurements provide valuable morphological information, they do not capture changes in compressibility or elasticity—features that are often compromised in fat pad pathology. In symptomatic or degenerative states, the fat pad may exhibit increased stiffness, reduced deformation capacity, or fragmentation of its elastic septa.

Regarding sex, there were no statistically significant difference between the PFP of men and women. Despite this, it is possible to observe that adipose tissue in men were consistently thicker in all metatarsal heads.

BMI showed a positive correlation only with the PFP beneath the fourth metatarsal head, although a similar trend was noted in the central forefoot region (second and third metatarsal heads). These results highlight the importance of considering both the morphological characteristics of the PFP and individual anthropometric factors in clinical and biomechanical forefoot assessment. However, this result must be taken with caution given that this was the only significant finding among multiple comparisons, and there is a possibility that there may have been a Type I error.

In agreement with these findings, Chih-Chin Hsu et al. [27] reported, in patients with type 2 diabetes mellitus, a correlation between BMI and the energy dissipation ratio (EDR) at the fourth metatarsal head, without significant associations in the remaining metatarsals. The consistency of this localization suggests that the fourth metatarsal head may represent an anatomically sensitive region to BMI-related variations, both in healthy individuals and in populations with metabolic disorders. In our study, increased PFP thickness may reflect a compensatory structural adaptation to greater mechanical load, whereas in Hsu et al.’s work variations in EDR may be related to viscoelastic changes induced by diabetes. Overall, these findings support the hypothesis that BMI exerts a localized influence on the structural and functional properties of the plantar tissue, with the region of the fourth metatarsal head being particularly relevant in this adaptive response.

Previous studies, such as that by Abouaesha et al. [25], also found a positive association between BMI and total plantar tissue thickness (PFP plus skin) in an older population of diabetic patients with neuropathy. That study showed that individuals with higher BMI presented thicker PFP and, consequently, lower plantar pressures. Future studies with larger and more diverse samples, including a balanced representation of all BMI categories, may provide a more accurate understanding of this relationship.

As for the limitations of the present study, the relatively moderate sample size (35 participants) and the inclusion of only healthy individuals should be considered. It would be valuable to conduct similar analyses in a comparable sample of young subjects with metatarsal pathology to compare both groups. As a strength of the present study the methodological approach adopted may serve as a preliminary reference for future research by providing normative data on the thickness of the subcutaneous tissue beneath the five metatarsal heads. These findings highlight the importance of continuing to investigate this parameter to determine whether PFP thickness plays a protective role in the prevention, since a reduced plantar fat pad thickness under the metatarsal heads may decrease the foot’s ability to absorb impact, increasing plantar pressure and risk of forefoot pain or pathology.

The results obtained from young and healthy individuals provide valuable information for clinical practice and research within the field of healthcare. Establishing normative values for PFP thickness enables the identification, in later stages of life, of deviations potentially associated with aging or metabolic disorders, thereby reinforcing a preventive approach to health care. Furthermore, understanding PFP characteristics in young populations can serve as a foundation for educational and foot health promotion programs, supporting early interventions aimed at maintaining functionality and preventing injuries.

## 5. Conclusions

The central forefoot region, corresponding to the second and third metatarsal heads, exhibits the greatest PFP thickness, with no significant differences observed between healthy young men and women. This finding suggests that the distribution of plantar adipose tissue in this region follows a relatively constant anatomical pattern, independent of sex. Furthermore, increased BMI appears to exert a moderate influence on the thickness of the tissue beneath the fourth metatarsal head, which may represent a potential protective mechanism against the development of overload-related forefoot pathology.

## Figures and Tables

**Table 1:** Anthropometric characteristics of the sample.

Variable	Overall (n = 35)	Men (n = 17)	Women (n = 18)
	Mean ± SD (Range)	Mean ± SD (Range)	Mean ± SD (Range)
Age (years)	22.71 ± 2.27 (18–27)	22.35 ± 2.28 (18–27)	23.05 ± 2.26 (20–27)
Height (cm.)	169.63 ± 8.91 (157–184)	177.23 ± 5.82 (184–160)	162.44 ± 3.79 (157–170)
Weight (kg.)	69.86 ± 11.42 (51.3–91)	78.24 ± 7.89 (66–91)	61.95 ± 8.11 (51.3–84)
BMI (kg/m^2^)	24.19 ± 2.89 (19.8–32)	24.98 ± 2.68 (20.4–28.9)	23.48 ± 2.97 (19.8–32)

**Table 2:** Descriptive statistics of plantar fat pad thickness under the metatarsal heads.

PFP Thickness	Minimum (mm)	Maximum (mm)	Mean (mm)	Standard Deviation
1st MTH	4.4	8.6	6.6	1.0
2nd MTH	4.7	8.5	7.1	0.9
3rd MTH	4.8	8.8	6.9	0.9
4th MTH	4.8	8.0	6.8	0.9
5th MTH	4.5	8.8	5.8	0.9

**Table 3:** Plantar fat pad thickness by sex.

PFP Thickness	Sex	Mean (mm)	Standard Deviation	*p*
1st MTH	Woman (n = 18)	6.2	0.8	0.283
	Men (n = 17)	7.0	1.0	
2nd MTH	Woman (n = 18)	6.8	0.7	0.124
	Men (n = 17)	7.5	0.9	
3rd MTH	Woman (n = 18)	6.5	0.8	0.724
	Men (n = 17)	7.3	0.8	
4th MTH	Woman (n = 18)	6.5	0.9	0.500
	Men (n = 17)	7.1	0.8	
5th MTH	Woman (n = 18)	5.6	0.7	0.781
	Men (n = 17)	6.0	1.0	

**Table 4:** Correlations between body mass index and plantar fat pad thickness.

Variable 1	Variable 2	Pearson’s r	*p*
BMI	PFP thickness 1st MTH	0.186	0.284
BMI	PFP thickness 2nd MTH	0.314	0.067
BMI	PFP thickness 3rd MTH	0.315	0.066
BMI	PFP thickness 4th MTH	0.358	0.035
BMI	PFP thickness 5th MTH	0.154	0.377

## Data Availability

The raw data supporting the conclusions of this article will be made available by the authors on request.
